# Differential Expression of Carotenogenic Genes, Associated Changes on Astaxanthin Production and Photosynthesis Features Induced by JA in *H. pluvialis*

**DOI:** 10.1371/journal.pone.0042243

**Published:** 2012-08-01

**Authors:** Zhengquan Gao, Chunxiao Meng, Xiaowen Zhang, Dong Xu, Yuefeng Zhao, Yitao Wang, Hongxin Lv, Liming Yang, Lingling Chen, Naihao Ye

**Affiliations:** 1 State Key Laboratory of Agricultural Microbiology, College of Life Science and Technology, Huazhong Agricultural University, Wuhan, Hubei, China; 2 School of Life Sciences, Shandong University of Technology, Zibo, Shandong, China; 3 Yellow Sea Fishery Research Institute, Chinese Academy of Fishery Sciences, Qingdao, Shandong, China; 4 Qingdao Agricultural University, Qingdao, Shandong, China; University of Connecticut, United States of America

## Abstract

*Haematococcus pluvialis* is an organism that under certain conditions can produce astaxanthin, an economically important carotenoid. In this study, the transcriptional expression patterns of eight carotenogenic genes of *H. pluvialis* in response to jasmonic acid (JA) were evaluated using real-time PCR. Astaxanthin accumulation action and photosynthesis flourescence were monitored at the same time. The results showed all eight genes exhibited higher transcriptional expression significantly under JA treatments. JA25 (25 mg/L) induction had greater effect (>10-fold up-regulation) on the transcriptional expression of *pds*, *crt*R-B and *lyc* than on *ipi*-1, *ipi*-2, *psy*, *bkt*2, and *cr*tO. JA50 (50 mg/L) treatment had greater impact on the transcriptional expression of *ipi-*1, *ipi-*2, *psy*, *crt*R-B and *cr*tO than on *pds*, *lyc* and *bkt*2. Astaxanthin biosynthesis in the presence of JA appeared to be up-regulated mainly by *psy*, *pds*, *crt*R-B, *lyc*, *bkt*2 and *cr*tO at the transcriptional level and *ipi-*1, *ipi-*2 at both transcriptional and post-transcriptional levels. Under JA induction, the photosynthetic efficiency [Y (II)] and the maximum quantum efficiency of PS II (Fv/Fm) decreased significantly, but the non-photochemical quenching of chlorophyll fluorescence (NPQ) increased drastically with the accumulation of astaxanthin.

## Introduction

Astaxanthin (3,3′-dihydroxy-β,β-carotene-4,4-dione) is a red ketocarotenoid used as a pigmentation source in aquaculture and the nutraceutical, pharmaceutical, and cosmetic industries [1]. The freshwater unicellular alga *Haematococcus pluvialis* is a good astaxanthin-producing organism since it can accumulate astaxanthin up to 4% of dry weight [2]. *H. pluvialis* synthesises abundant astaxanthin in response to various stress conditions, such as high light, salinity, acetate addition [3], nutrient stress [4], and high carbon/nitrogen ratio [5].

The pathway of astaxanthin synthesis in *H. pluvialis* had been clarified using specific inhibitors [6]. The following carotenoid biosynthesis genes have been cloned and characterized in *H. pluvialis*: they are isopentenyl diphosphate isomerase gene (*ipi*) [7], phytoene synthase gene (*psy*) [8], phytoene desaturase gene (*pds*) [6], [9], lycopene β cyclase gene (*lyc*) [8], β-carotenoid oxygenase gene (*crt*O) [10], three carotenoid ketolase genes (*bkt*s) [10], and carotenoid hydroxylase gene (*crt*R-B) [11]. Considerable efforts have been focused on understanding regulation of carotenogenic genes related to astaxanthin biosynthesis in *H. pluvialis* and on the physiological role of astaxanthin in the response to various stresses. The mechanisms for these processes remain unclear, although most phycologists believe that astaxanthin accumulation is a defensive reaction of *H. pluvialis* to stresses.

Carotenogenesis during stress-induced accumulation of astaxanthin is also well documented by studying the transcriptional expression changes of carotenogenic genes under various stress conditions [6], [7], [8], [10], [12], [13]. Our previous studies revealed that the 5′-flanking regions of some key genes involved in astaxanthin biosynthesis, including *bkt* and *crt*O might contain jasmonic acid (JA)-responsive element [14], [15]. Lu et al. also reported that the 5′-flanking regions of the genes *bkt*2 and *bkt*3 contain regulatory element that are sensitive to methyl jasmonate (MJ), but its regulatory mechanism remains undefined [16].

Many plant secondary metabolites may be induced by JAs via up-regulating the expression of a series of key enzyme genes when plants are under stressed or treated by exogenous JA [17], [18]. For example, the caffeoylputrescine content of tomato seedlings was increased when treated with JA [19]. JA also promoted nicotine biosynthesis in transgenic tobacco by causing over-expression of allene oxide cyclase from *Hyoscyamus niger* [20]. These results suggest that JAs could be used as an effective regulator to stimulate astaxanthin production in *H. pluvialis*.

Carotenoids can act as accessory light-harvesting pigments and perform an essential photoprotective role by quenching triplet state chlorophyll molecules and scavenging toxic oxygen [21]. Although some photoprotective mechanisms have been proposed, the actual photoprotective function of astaxanthin in red cysts of *H. pluvialis* remains poorly understood [22].

The goals of this study were to investigate the transcriptional expression patterns of carotenogenic genes and accumulating astaxanthin under treatment of exogenous JA in *H. pluvialis*, and attempt to evaluate the relationship between JA-induced astaxanthin accumulation and photoprotection in *H. pluvialis*. The results will provide new insight into the multifunctional roles of carotenogenesis in response to JA induction. In order to assess the impact of JA on *H. pluvialis*, the transcriptional expression of eight carotenogenic genes (*ipi*-1, *ipi*-2, *psy*, *pds*, *lyc*, *crt*R-B, *bkt*2 and *cr*tO) during astaxanthin biosynthesis was analyzed using real-time PCR. The non-photochemical quenching of chlorophyll a fluorescence (NPQ), photosynthetic efficiency [Y (II)], and the maximum quantum efficiency of PS II (Fv/Fm) of samples and concentration of astaxanthin were also measured.

## Materials and Methods

### Algal Strain and Growth Conditions

*H. pluvialis* strain 712 was obtained from Institute of Oceanology, Chinese Academy of Sciences. Samples of *H. pluvialis* were grown in MCM medium [24] and cultured in 1000 mL erlenmeyer flasks which were placed in an illuminating incubator (Ningbo Jiangnan Instrument Factory, GXZ-380, Ningbo, China) under a light intensity of 25 µmol photons m^–2^s^–1^ on a 12 h: 12 h light/dark cycle at 20°C without aeration. All flasks were shaken manually twice at fixed time every day.

### JA Treatment

*H. pluvialis* algae in the logarithmic phase were divided into three treatments with 1.25×10^5^ cells final content to the control (0 mg/L JA) and two JA treatment groups, JA25 (25 mg/L JA) and JA50 (50 mg/L JA). An equal amount of ethanol was added to the control [17]. After the JA solution was added, the algal cells were harvested at regular intervals over the course of 18 days.

### Measurement of Astaxanthin Content

The astaxanthin was extracted and analyzed following Boussiba and Vonshak [23]. The major absorption peak in dimethylsulfoxide is at ca. 490 nm, and astaxan– thin concentration can be calculated according to formula: C(mg/L) = (4.5×*OD_490_*×*Va*)/*Vb*. *Va* and *Vb* represented volume of dimethylsulfoxide and microalgae samples, respectively). Equal aliquots of culture from each treatment and the control were harvested at different time points and lyophilized. Lyophilized cells were then extracted with dimethylsulfoxide repeatedly until the pellet became colorless. Absorbance of the extracts was read at 490 nm with a spectrophotometer (T6 new century, Beijing General Instrument Ltd, China). The blank contained dimethylsulfoxide only.

### Optical Microscopy

Optical microscope observations were made of the morphology, color, biomass and pigment accumulation of *H. pluvialis* and were performed using an optical microscope (Nikon Eclipse 80i microscope, Nikon, Tokyo, Japan). Cell number was counted microscopically using hemocytometer. At least 150 cells and six separate slides were evaluated per sample.

### RNA Isolation and RT-PCR

Samples were frozen in liquid nitrogen and then ground into a fine powder. The total RNA was subsequently extracted using Trizol reagent according to the manufacture’s instructions and dissolved in diethypyrocarbonate treated water. In this protocol, RNA was digested with DNaseI. The cDNA used for real-time PCR was synthesized from total RNA using Moloney murine leukemia virus reverse transcriptase (Promega Biotech Co., Madison, WI, USA).

The gene-specific primers for the eight genes were designed using Primer 3 (Table 1) software and synthesized (BGI, China). The reaction mixture contained 14.5 µL pure water, 2 µL 10×PCR buffer (Mg^2+^ plus), 0.4 µL dNTP (10 mM·L^−1^), 1 µL (10 µM·L^−1^) of each primer, and 0.2 µL rTaq DNA Polymerase (TaKaRa, Dalian, China). Amplification was conducted by subjecting the samples to the following conditions: initial denaturation at 94°C for 4 min followed by 33 cycles of 94°C for 40 s, 55°C (*psy*, *crt*O, *act*)/58°C (*pds*)/61°C (*bkt*2, *lyc*, *ipi*-1)/62°C (*ipi*-2, *crt*R-B) for 40 s, and 72°C for 2 min, with a final extension at 72°C for 7 min. The PCR products were then resolved by electrophoresis on 1% agarose gel, after which the fragment of interest was excised, purified using an agarose gel DNA fragment recovery kit (TaKaRa), cloned into PMD-18T vector (TaKaRa) and sequenced (BGI, China). Each sequence was examined for homology with known sequences using the BLAST program available at the National Center for Biotechnology Information website (http://www.ncbi.nlm.nih.gov/blast).

**Table 1:** Gene-specific primers and annealing temperatures used for qRT-PCR.

Primer	Primer sequence (5′–3′)	Annealing temperature(°C)	GenBank ID
*psy*F	CGATACCAGACCTTCGACG		
*psy*R	TGCCTTATAGACCACATCCAT	55	AF305430
*pds*F	ACCACGTCGAAGGAATATCG		
*pds*R	TCTGTCGGGAACAGCCG	58	X86783
*lyc*F	TGGAGCTGCTGCTGTCCCT		
*lyc*R	GAAGAAGAGCGTGATGCCGA	61	AY182008
*crt*R-BF	ACACCTCGCACTGGACCCT		
*crt*R-BR	GTATAGCGTGATGCCCAGCC	62	AF162276
*bkt*2F	CAATCTTGTCAGCATTCCGC		
*bkt*2R	CAGGAAGCTCATCACATCAGAT	61	AY603347
*ipi*-1F	GCGAGCACGAAATGGACTAC		
*ipi*-1R	GCTGCATCATCTGCCGCA	61	AF082325
*ipi*-2F	AGTACCTGGCGCAAAAGCTG		
*ipi*-2R	GTTGGCCCGGATGAATAAGA	62	AF082326
*crt*OF	ACGTACATGCCCCACAAG		
*crt*OR	CAGGTCGAAGTGGTAGCAGGT	55	X86782
*act*F	TGCCGAGCGTGAAATTGTGAGG		
*actR*	CGTGAATGCCAGCAGCCTCCA	55	Huang et al, 2006

### Real-time Quantitative PCR Analysis

Real-time fluorescence quantitative PCR was used to investigate the expression kinetics of the eight carotenoid genes simultaneously in response to JA treatment. The actin gene was used as a reference for total RNA. For real-time PCR, pairs of gene-specific primers were designed using GenBank data. PCR products were then quantified continuously with the ABI StepOne Plus Real-Time PCR System (Applied Biosystems, USA) using SYBR green fluorescence (Takara) according to the manufacturer’s instructions. The PCR amplification profile was 95°C for 30 s followed by 40 cycles of 95°C for 5 s, 55°C (*psy*, *crt*O, *act*)/58°C (*pds*)/61°C (*bkt*2, *lyc*, *ipi*-1)/62°C (*ipi*-2, *crt*R-B) for 15 s, and 72°C for 35 s. The 2^−ΔΔCT^ method [24] was used to analyze quantitative real-time PCR data.

### Photosynthesis Measurements

The Fv/Fm, Y (II) and NPQ of fresh alga cultures were measured using the pulse-amplitude modulated method on a Dual-PAM-100 (Walz, Effeltrich, Germany) connected to a PC running WinControl software The details regarding the operating parameters for the variable fluorescence measurements are as follows: wavelength: 620nm, irradiance of the measuring light: 12 µmol photons m^–2^s^–1^, actinic light: 126 µmol m^–2^s^–1^. Before measurement, the samples were kept in the dark for 15 min and the original fluorescence (*F*_0_) was determined under low measuring light. A saturating light pulse was applied to obtain maximum fluorescence (*F*_m_) in the dark-adapted samples. The *F*_m_ yield in illuminated samples is denoted as *F*_m_’, and *F*_t_ is real-time fluorescence yield. All experiments were conducted in triplicate. The effective PS II quantum yield was calculated as follows:

*NPQ = *(*Fm* − *Fm*′)/*Fm*′

*Y(II) = *(*F*_m_′ – *F*_t_)/*F*_m_′.

*Fv*/*Fm = *(*Fm* − *Fo*)/*Fm*

### Statistical Analysis

The means ± SD were derived from all data and were statistically analyzed with one-way ANOVA (SPSS 17.0). LSD multiple comparisons test were used to test the differences among groups of different trials. *p*-values of less than 0.05 and 0.01 were considered to be statistically significant and extreme significant, respectively.

## Results

In order to better understand the regulatory underpinnings of JA induced *H. pluvialis* accumulating astaxanthin, the growth curves of alga cells, astxanthin concentration, photosynthesis flourescence and the transcriptional expression patterns of eight carotenogenic genes were studied in our experiment.

### JA-induced Astaxanthin Accumulation in H. Pluvialis

**Figure 1:**
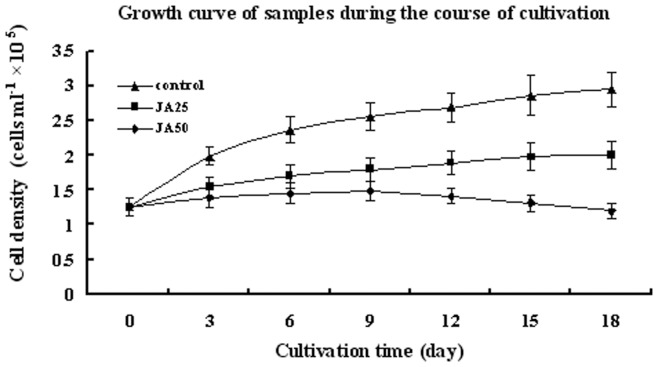
The growth curves of treatments and control during the course of cultivation.

**Figure 2:**
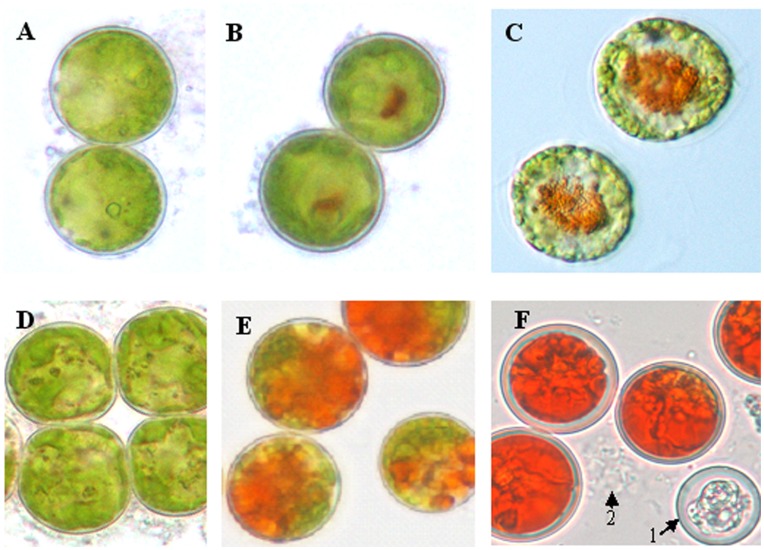
Microscopic images (400×) of *H. pluvialis* cells culture samples day 3 and 18 after treatment with JA. (A), (B), (C) represent the control, JA25 sample and JA50 sample after 3 days of treatment, respectively; (D), (E), (F) represent the control, JA25 sample and JA50 sample after 18 days of treatment, respectively; arrow 1 and arrow 2 show the algae cells were whitened or autolysed in the JA50 samples.

**Figure 3:**
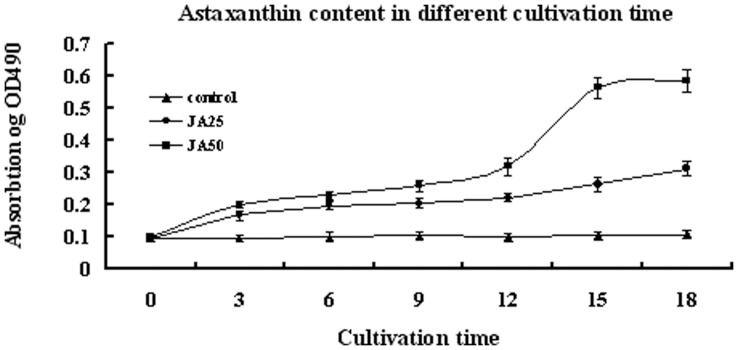
Astaxanthin accumulations of control, JA25 sample and JA50 sample during cultivation. OD_490_ represents relative astaxanthin content in alga culture solution.

Figure 1 shows the growth curves of treatments and control. Algae cells of control grow commonly but decelerate in the later period of incubation. However, growth and breed of samples in treatments were suppressed significantly (p<0.05). Microscopy observations revealed the initial color change from green to red on day 3 after application JA in both treatments (Figure 2A, Figure 2B, Figure 2C). On day 18, about 45% of the algae cells were whitened or underwent autolysis in the JA50 samples. The percentage of red cells was about 98% of total unwhitened cells in JA50 and 50% of total algae cells in JA25 treatments, respectively (Figure 2D, Figure 2E, Figure 2F). On day 18, JA50 treatments resulted in the reddest culture, followed by JA25 treatment. However, JA25 treatment resulted in the highest astaxanthin production (1.458 mg/L alga culture solution, p<0.01 compared with control and JA50), followed by JA50 treatments (1.129 mg/L alga culture solution, p<0.01 compared with control and JA25) and the control (0.0468 mg/L alga culture solution) (Figure 3).

### Photosynthesis Measurement

**Figure 4:**
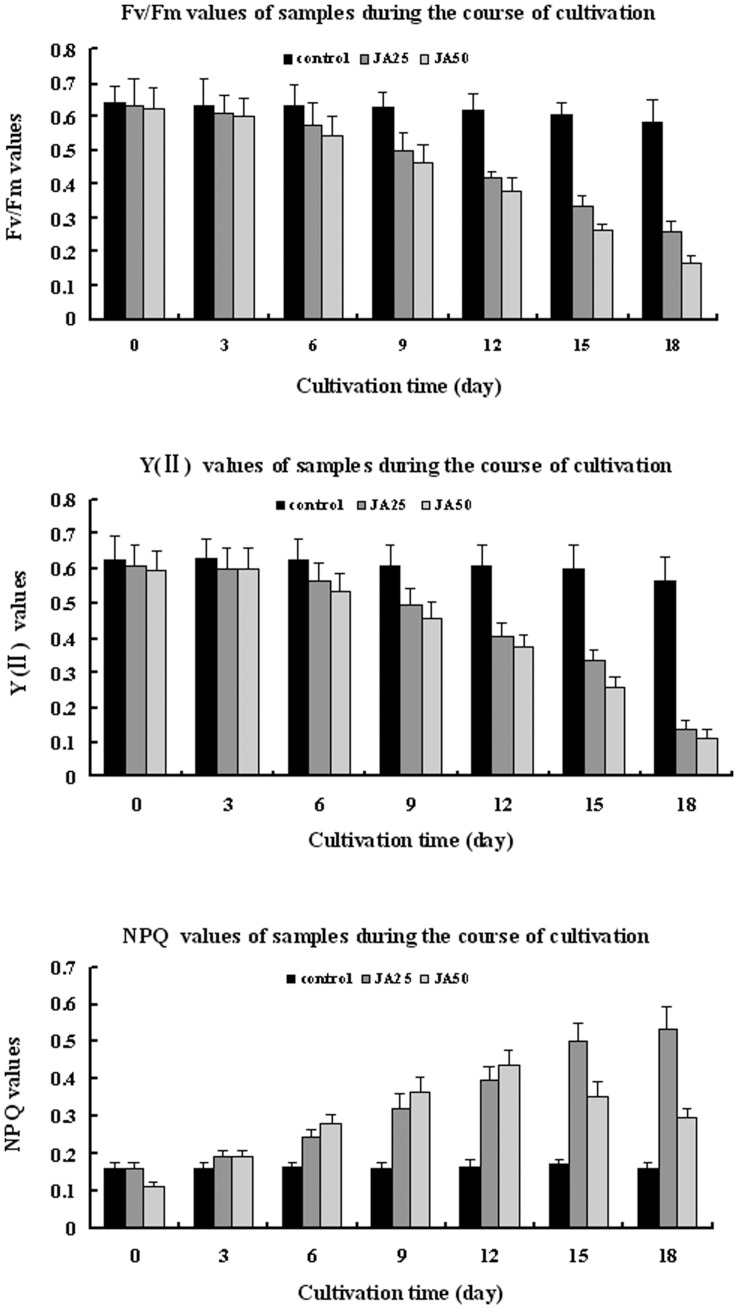
Changes of three photosynthesis fluorescence parameters of control, JA25 sample and JA50 sample on day 0, 3, 6, 9, 12, 15, 18 of incubation. (A), (B), (C) represent changs of Fv/Fm, Y (II) and NPQ, respectively.

Fv/Fm, Y (II) and NPQ were measured to evaluate changes in the photosynthesis efficiency and photoprotection capacity under induction of JA. The Fv/Fm, Y (II) and NPQ differed among the three samples during the course of incubation. The control showed no distinct change in NPQ and slight decrease in Fv/Fm and Y (II). Fv/Fm of the JA25 and JA50 treatments decreased by 59.1% and 73.1% after treatment of 18 days, respectively (p<0.01) (Figure 4A); Y (II) of the JA25 and JA50 treatments decreased by 77.6% and 80.9%, respectively (p<0.01) (Figure 4B); and NPQ of the two treatments increased 4.45- and 2.68-fold compared to the control, respectively (p<0.01) (Figure 4C). Thus, JA treatment led to accumulation of astaxanthin, decreased photosynthesis and increased photoprotection.

### Patterns of JA-induced Transcription of Carotenogenic Genes

**Figure 5:**
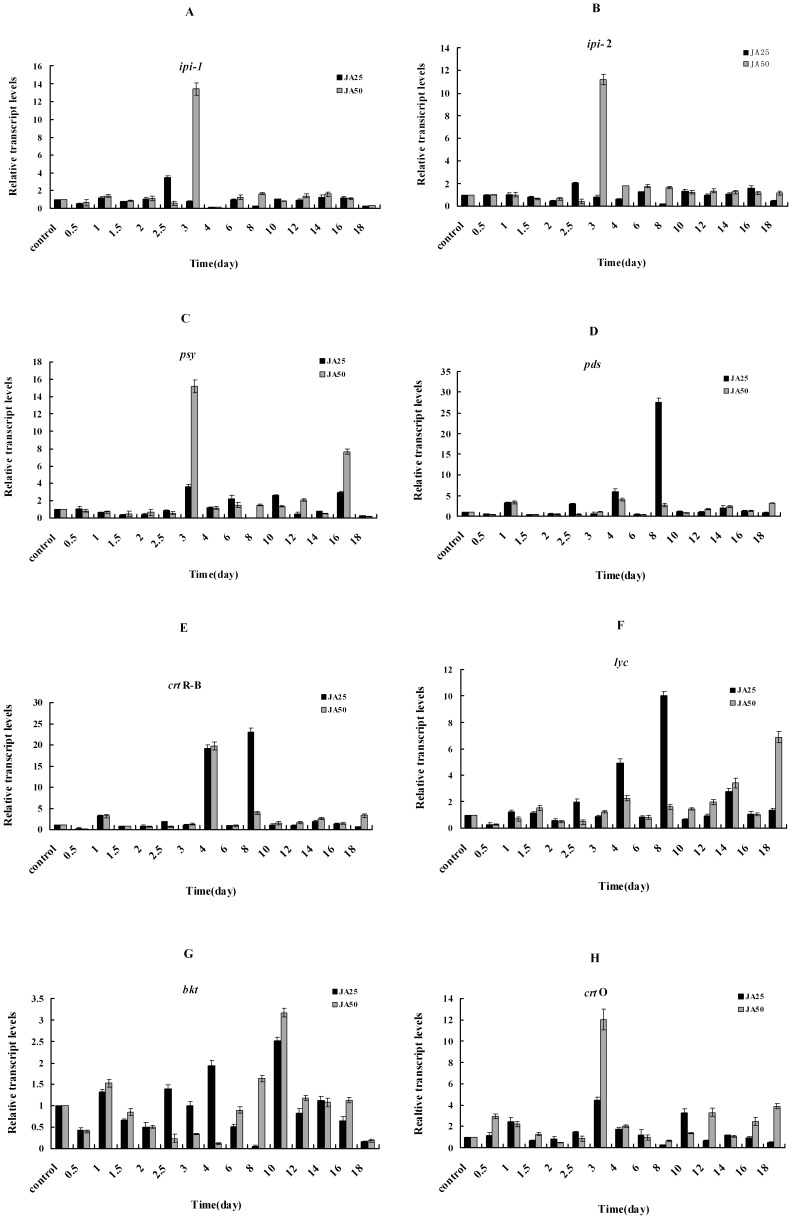
The effects of JA on the transcript levels expression kinetics of eight carotenogenic genes in *H. pluvialis* during incubation. (A), (B), (C), (D), (E), (F), (G) and (H) represent transcript levels expression kinetics of *ipi*-1, *ipi*-2, *psy*, *pds*, *crt*R-B, *lyc*, *bkt*2 and *crt*O respectively.

For *ipi*-1 and *ipi*-2, an initial increase and the maximum transcriptional level occurred on day 2.5 in the JA25 treatment, with 3.4- and 2.0-fold higher than that of control, respectively. The highest transcriptional level of *ipi*-1 and *ipi*-2 in the JA50 treatment occurred on day 3, with 13.4- and 11.2-fold higher levels compared to the control (p<0.01), respectively. Then they declined sharply until day 18 (Figure 5A and Figure 5B).

The initial increase and maximum transcriptional expression of *psy* in both the JA25 and JA50 treatments occurred on day 3, and the levels were 3.6- and 15.2-fold higher than that of control, respectively (p<0.01); after day 3 the levels showed an irregular descending trend until day 18 (Figure 5C).

In both treatment groups, the first peak of transcriptional level of *pds* (3.2-fold higher than that of the control) occurred on day 1, whereas the highest *pds* steady-state mRNA transcriptional level occurred on day 8 (27.5-fold higher than control, p<0.01) in the JA25 treatment, and on day 4 (4-fold higher than the control, p<0.01) in the JA50 treatment. After these time points, a similar decreasing trend in the *pds* transcriptional level was observed in both treatment groups until day 18 (Figure 5D).

The patterns observed from *crt*R-B were similar to those observed for *pds*. The initial increased expression of *crt*R-B was 4.0-fold higher than that of the control on day 1 in both JA treatments. The maximum transcriptional levels of *crt*R-B occurred on day 8 and 4 (23- and 20-fold increases, p<0.01) in the JA25 and JA50 treatments, respectively, then they declined sharply (Figure 5E).

The initial enhanced expression of *lyc* occurred on day 2.5 and 4 in the JA25 and JA50 treatments, with 2.0-fold higher than that of the control, respectively. The maximum transcriptional level of *lyc* in the JA25 and JA50 treatments occurred on day 8 and 18, respectively, with 10- and 6.9-fold increases, respectively (p<0.01). After day 8 in the JA25 treatment, the *lyc* level declined, whereas an irregular increasing trend was detected in the JA50 treatment throughout the experiment (Figure 5F).

JA treatment seemed to have a less significant effect on *bkt*2 expression than the other seven genes. The *bkt*2 reached its highest transcriptional expression on day 10 in both treatments; they were only 2.5- and 3.2-fold higher than that of the control in the JA25 and JA50 treatments, respectively (p<0.01) (Figure 5G).

In the JA25 treatment, the initial and highest transcriptional levels of *crt*O occurred on day 1 and 3, respectively with 2.5- and 4.5-fold higher than that of the control (p<0.01), respectively. In the JA50 treatment, the *crt*O transcriptional level rose to 3.0-fold higher than the control on day 0.5, then declined, and then reached its highest level (12.0-fold increase) on day 3 (p<0.01) (Figure 5H).

## Discussion

JA is the quickest signal molecule, a critical component of the complex signaling networks in plants defense responses [25]–[27], and a key player in regulating abiotic stress-induced gene expression in higher plants [28], [29]. JA has been found in algae, but information about its mechanisms of action and role in regulating secondary metabolites is scarce [26]. In *Fucus*, JA was reported to trigger defense responses after plant damage caused by phytophages [30]. Higher concentrations of MJ (500 µM) were found to inhibit astaxanthin accumulation, whereas lower concentrations (10 µM) could be used to elicit secondary carotenoid production. The results of current study indicated that the astaxanthin concentrations of two JA treatments were much higher than that of control after 18 days of culture. However, the astaxanthin production of the JA50 samples was lower than that of the JA25 samples, although the algae cells in the former were much redder than those in the latter under microscope. This phenomenon is explained that the cell density of the JA25 sample was thicker than that of the JA50 because of autolysis caused by the JA50 treatment. Overall, these results illustrated that exogenous JA could induce *H. pluvialis* to accumulate astaxanthin effectively.

Carotenoids play an important role in the dissipation of excess light intensities. However, the photoprotective role of astaxanthin in *H. pluvialis* is not well clarified at present. Several researchers have attempted to elucidate the role of astaxanthin as a protector against high irradiation. Tan et al. postulated that the decline of O_2_ evolution in red cells is largely attributable to respiration rate increase, the impairment of linear electron flow from PS II to PS I, and decrease in components of the photosystems [31]. Wang et al., however, reported that astaxanthin absorbed light in the blue region, protected the photosynthetic apparatus, and served as a physicochemical barrier to protect the replicating DNA from oxidation as the cells divide [22]. Lemoine and Schoefs posited that astaxanthin synthesis was not only a photoprotective response to stress but also a multifunctional response triggered by factors such as high light level, nutrient starvation, or the presence of phytohormones. This multifunctional response would help cells cope with oxidative stress [32]. The ways that astaxanthin protect photosynthetic apparatus is a controversial issue, but astaxanthin can serve as a protector photosynthetic system is a indisputable fact according to references above. The Y(II) and Fv/Fm results of the present study agreed with those reported by Fan et al. [33] but differed from those of Hagen et al. [34]. NPQ results demonstrated that the accumulation of astaxanthin was directly related to the augmented photoprotection, which confirmed the photoprotective role of astaxanthin in *H. pluvialis* one more time.

Many studies have reported induction of carotenogenic genes expression and increase in total carotenoid under various stresses in *H. pluvialis*. The results of transcription expression of *ipi*, *psy*, *pds*, *crt*O, *crt*R-B, and *lcy* suggested that individual carotenogenic genes underwent transient up-regulation in response to increased light density. [6], [7], [8], [10], [35]. in *H. pluvialis* under high photon flux density (PFD) The higher transcriptional levels of *psy* and *crt*R-B were correlated with higher astaxanthin concentrations, suggesting that astaxanthin biosynthesis is under the tight transcriptional control of *psy* and *crt*R-B [13], [35], which was in accord with our present results (Figure 5C and 5E). Lu et al. also found that exogenous MJ (2 and 20 mg/L) increased the production of astaxanthin and the transcriptional expression of three *bkts* of *H. pluvialis* [16]. Grünewald et al. demonstrated regulation of *pds* at the transcriptional level in *H. fluvialis* [6]. Li et al. concluded that astaxanthin biosynthesis is mainly regulated at the transcriptional level of *crt*R-B in of *H. pluvialis* [13]. These results were also in line with our results in Figure 5.

The present study is the first report attempting to describe the effects of JA on the expression kinetics of eight carotenogenic genes and expound the regulation of JA on them in *H. pluvialis.* The research was elementary and much more research works are essential before the regulatory mechanism of JA is clarified thoroughly in *H. pluvialis*, such as screening differentially expressed genes and regulatory factors using transcriptome sequencing, identifying the differential expression proteins under JA induction and determining the relationship between carotenoid biosynthesis genes and these proteins. However, this paper confirmed the previous conjecture presented by Meng et al. [15], [16], [36] and revealed several new aspects of carotenogenic genes expression kinetics:

1. Astaxanthin accumulation by *H. pluvialis* was induced effectively by JA. High levels of astaxanthin accumulation are likely mainly due to the up-regulation of carotenogenic genes.
2. Carotenogenic genes exhibited different expression profiles when exposed to the same concentration of JA, although expression of all eight genes was up-regulated during treatment course. Results showed that JA25 induction had a greater effect on the transcriptional expression of *pds*, *crt*R-B and *lyc* (>10- fold up-regulation) than on *ipi*-1, *ipi*-2, *psy*, *bkt*2, and *cr*tO. JA50 treatment had a greater impact on the transcriptional expression of *ipi-*1, *ipi-*2, *psy*, *crt*R-B and *cr*tO than on *pds*, *lyc* and *bkt*2.
3. Six carotenogenic genes (*ipi-*1, *ipi-*2, *psy*, *cr*tO, *lyc*, and *bkt*2) exhibited the same expression profiles but the other two ones (*pds* and *crt*R-B) with different expression profiles when exposed to different concentration of JA. The correlation between maximum transcript levels of carotenogenic genes and the timing of astaxanthin accumulation is suggestive to the regulatory mechanism at work: For those genes which maximum transcriptional level preceded the time point of accumulating astaxanthin quickly were considered as likely were involved in controlling astaxanthin accumulation at post-transcriptional level; on the contrary, for other genes which transcriptional peaks lagged behind this time point, and they likely regulated astaxanthin accumulation at transcriptional level [13], [35]. According to this standard, *ipi-*1 and *ipi-*2 might up-regulate astaxanthin accumulation at post-transcriptional level and *psy*, *cr*tO, *pds*, *crt*R-B, *lyc* and *bkt*2 might up-regulate at transcriptional level in the JA25 treatment. The transcriptional expression of the JA50 samples varied from JA25 treatments. Based on the criterion above, all the eight carotenogenic genes might up-regulate astaxanthin accumulation at transcriptional level. Since *ipi-*1, *ipi-*2 were up-regulated by JA25 at post transcriptional level and by JA50 at transcriptional level, which suggested that *ipi-*1, *ipi-*2 enhanced astaxanthin accumulation at both two molecular levels under different concentration of JA treatment.
